# Scientometric analysis of the forensic science literature for fibre as an evidence type: Access and data availability

**DOI:** 10.1016/j.fsisyn.2022.100269

**Published:** 2022-05-17

**Authors:** Virginie Galais, Holly Fleming, Niamh Nic Daéid, Hervé Ménard

**Affiliations:** Leverhulme Research Centre for Forensic Science, University of Dundee, Dundee, DD1 4HN, UK

## Abstract

The large volume of information available within citation databases has become a challenge to manage and distil in all areas of research. In this study, a scientometric approach has been applied to fibres as an evidence type using information contained in Scopus and Web of Science. A comparison was also made with the references listed in the INTERPOL International Forensic Science Managers Symposium Science (IFSMS) reports (2004–2019) where only a limited number of documents were common with the citation databases, illustrating the value of the IFSMS reports. Finally, this study also highlights that data availability and location are generally omitted in publications. The forensic science community has an opportunity to change this culture and lead the way in making their data available, aligning with the ideals of fairness, openness and transparency of the underpinning data upon which scientific developments are based.

## Funding information

This research was funded by the 10.13039/501100000275Leverhulme Trust RC-1015-011.

## 1 Introduction

Literature reviews and studies that synthesise previous knowledge are essential to developing an understanding of a field of research and how it may be enhanced by identifying potential information gaps. References are used to populate literature studies and are generally provided by citation databases such as Scopus, PubMed, Google Scholar or Web of Science. The use of such databases as repositories for research papers has increased in recent decades, however and as an unintended consequence, the increasing number of publications for specific topics can be problematic to deal with in a way that enables a depth of knowledge and a relationship based understanding of the domain under study. One possible approach to address this issue is to analyse the entire body of research and output generated from a field of study using bibliometrics and scientometric approaches.

Scientific indexing started in the early 1900s with for example the first publication of the Chemical Abstracts in 1907 by the American Chemical Society, and with continuous growth in the number of research outputs, various ordering and classification methods were subsequently developed. The most commonly used relationships include; Lotka's law of scientific productivity [1], Zipf's law of word occurrence [2] and Bradford's law of scattering [3]. The application of these and other methods have led to the definition of terms now associated with the analysis of the literature and the information it encompasses. One such term is bibliometrics, often credited to Pritchard, who described it as “*the application of mathematical and statistical methods to books and other media of communication*” [4]. Another well-known term generally presented as a synonym of bibliometric is scientometric [5], and further historical discussion on the term “scientometric” can be found in [6]. Bibliometric and scientometric methods are closely related and often indistinguishable as they both follow the same concepts; however, they differ in how they are attributed: bibliometrics is attributed to library and document science while scientometric is attributed to the science of science.

Many studies have reported research metrics for different databases and the decision to select one specific database to perform a bibliometric analysis may be due to the research topic, discipline, the requested information, or the accessibility of the research publications (see for example [7,8]). Although several different databases provide information on the documents they contain, there is no universal answer as to which citation database to use for publication searches and analysis of any given topic. The most commonly known citation databases are Scopus, Web of Science, Google Scholar and PubMed, and several comparison studies have been reported evaluating them against each other; see for example Falagas et al. [7] or Harzing and Alakangas [9]. Other citation databases were evaluated by Gusenbauer and Haddaway [8]. As well as expanding accessible materials, the content of the databases can also be discipline specific. PubMed primarily focuses on biomedicine and health science while Scopus, Web of Science, and Google Scholar are multidisciplinary. While the subject coverage is an essential factor in deciding on the selection of a citation database [10], the export capacity of the results is also of significance as this can be a limiting factor when dealing with large datasets. PubMed permits to download the details for a maximum of 10,000 references at once, Scopus allows a full export for up to 2000 references while for Web of Science it is just 500 references.

In forensic science, the selection of a citation database to retrieve information is dictated by the accessibility of the publications. Academic researchers generally have access to publications via their institutions' subscriptions while forensic science and other domain relevant practitioners may undertake literature searches and see a more limited range of materials. To facilitate and share the latest advancements in forensic research, reports from the INTERPOL International Forensic Science Managers Symposium (IFSMS) outline and summarise major areas of interest to forensic science practitioners across the INTERPOL member countries [11]. The information contained in the INTERPOL IFSMS reports can be seen as complementary to, for example, citation databases, but their extensive contents remain presented as literature reviews and these are challenging to process information from, see for example [12].

The application of publication metrics, currently not well known within the forensic science domain, is gaining interest with the development and improvement of citation databases and in particular are being viewed as critical tools in the exploration of relationships across the literature. The application of a scientometric approach to the scientific literature offers more objectivity than a traditional literature review [13]. Scientometric analysis is beginning to be progressively implemented in forensic science to present an overall analysis of the literature, initially by Sauvageau et al. [14] followed by Jesubright and Saravanan [15] and more recently for example by Raghunath [16], Sobreira et al. [17], Zolotenkova et al. [18], Liu [19] and Jones [20].

This work builds on these foundations and focuses on fibres generated from textiles and garments, using a bibliometric approach to survey the relevant forensic science literature where the search outputs of both Scopus and Web of Science are combined to generate a more comprehensive list of references.

## 2 Material and methods

### 2.1 Publication lists

The search query in the title, abstract and keyword for “Fibre OR Fiber” AND “Forensic” was made for both Web of Science and Scopus, for all years available until the end of 2020. English terms were used for querying the citation databases, but no attempt was made to exclude references written in another language. BibTex was selected as the export file format to facilitate the merging of the references using a script written in the R statistical programming language, RStudio (open source) and the library Bibliometrix. The code is available via a persistent identifier at https://doi.org/10.5281/zenodo.6363052 [21].

“Fibre” is not just limited to textile and garments but can also be associated with many academic disciplines such as medicine (muscle fibre) or physics (optical fibre), as well as a variety of types of forensic evidence such as drugs (herb fibres), questioned documents (paper fibres) or fingermarks (glass fibre brush), a categorisation based on field entries was included in the R script to filter out documents not associated specifically with the topic of interest.

Correction of titles and journals was carried out to identify duplicate entries between Scopus and Web of Science. Titles generated by Web of Science were used as a reference and if necessary, to correct those provided by Scopus, while the opposite approach was applied to the journal (source) title (e.g., Journal of Forensic Science) since in this case fewer inconsistencies were observed. To retain details, no attempt was made to correct journal titles that changed their name; for example, “Journal of the Forensic Science Society” becoming “Science and Justice” or “International Academy of Legal Medicine” having the previously title “Zeitschrift fur Rechtsmedizin”, but their respective results could be combined. When a reference was identified in both Scopus and Web of Science, the document type, affiliation, and source provided by Scopus was used by default. If the affiliation entry was missing for the output in Scopus, the output provided by Web of Science was used (if available).

The references listed for fibre evidence in the six most recent and digitally available International Forensic Science Managers Symposium (IFSMS) reviews covering 2004 to 2019, were used to create another dataset. Information found in these reports was manually compiled using materials available in citation databases such as Scopus, Web of Science, Google scholar or directly from their source (e.g., scientific journal, publisher, University libraries, etc.). If no information was retrieved for a specific reference, only the details listed in the IFSMS reports were used.

### 2.2 Keywords analysis

As Scopus and Web of Science generate specific keywords as part of their indexing systems which result in unrelated outputs, the keyword analysis carried out on these datasets considered only the available keywords provided by the authors of the relevant publication. The same choice was made for the IFSMS reports.

Available author's keywords (AK), chosen by the authors and associated with the manuscript at the time of submission, were used as part of the analysis. The author's keywords were corrected before analysis to remove spelling mistakes, regional variations, plurality, and synonyms of, for example, analytical techniques: in total 228 keywords were corrected and combined with a previous list of 447 corrected keywords published by Sobreira et al. [17]. This has also been used to address any spelling variation (fibre or fiber) associated to fibre as an evidence type, resulting in a more comprehensive list of 646 keywords once conflicts had been resolved*.* For display purposes and for all datasets (i.e., Scopus, Web of Science and the IFSMS reports) only keywords with an occurrence greater than or equal to 5 across the keywords list were included, (as such a keyword with an occurrence less than 5 would not appear in the figure).

### 2.3 Author analysis

167 author's names were corrected to resolve spelling mistakes, missing initials or the presence of diacritic characters (for example umlauts or β) that may induce errors following a change in text encoding (conversion to UTF-8 encoding). A correction was applied in the R script after manually checking conflicting names using other information in the dataset such as the author identification number (assigned by Scopus), affiliation and co-authorship. No attempt was made to distinguish between two authors with the same name, but no such conflict was observed during the analysis of authors covered here.

## 3 Results

### 3.1 Scopus and Web of Science dataset

A total of 1569 references were retrieved following a Scopus search using the keywords “TITLE-ABS-KEY ((Fibre OR Fiber) AND Forensic)”, and 867 from Web of Science using the same parameters. The document type distribution was found to be very similar between the two datasets, Table 1; with the majority of the outputs being research articles: 1194 (76.1%) for Scopus and 663 (76.5%) for Web of Science. Combining the two reference lists from Scopus and Web of Science produced a dataset containing 1756 references (Table 1) with 674 (38.4%) of those listed in both Scopus and Web of Science, 890 (50.7%) being exclusive to Scopus and 192 (10.9%) only indexed in Web of Science.

**Table 1:** Type of documents from Scopus and Web of Science for all years to 2020, IFSMS (2004–2019), covering a total of 1569 publications for Scopus, 867 for Web of Science, and 417 for the IFSMS reports. The % of Total represents the proportion of each document type for each database.

Document type	Scopus (% of Total)	Web of Science (% of Total)	Scopus and Web of Science combined dataset		IFSMS (% of Total)
			All topics and disciplines	Fibre, Textile	
Total	1569	867	1756	625	417
Articles	1194 (76.1)	663 (76.5)	1318 (75.1)	457 (73.1)	185 (44.4)
Book/Book chapter	80 (5.1)	19 (2.2)	86 (4.9)	45 (7.2)	19 (4.6)
Conference Material/Meeting Abstract/Proceedings paper	176 (11.2)	113 (13)	215^a^ (12.2)	85 (13.6)	90 (21.6)
Review	85 (5.4)	55 (6.3)	99 (5.6)	21 (3.4)	8 (1.9)
Retracted/Correction	1 (0.1)	1 (0.1)	2 (0.1)	–	–
Short survey	1 (0.1)	–	1 (0.1)	–	–
Editorial Material	2 (0.1)	6 (0.7)	3 (0.2)	1 (0.2)	–
Erratum	4 (0.3)	–	4 (0.2)		–
Letter	7 (0.4)	2 (0.2)	9 (0.5)	2 (0.3)	1 (0.2)
Note	17 (1.1)	8 (0.9)	17 (1)	14 (2.2)	1 (0.2)
Non-Specified	2 (0.1)	–	2 (0.1)	–	13 (3.1)
Thesis	–	–	–	–	3 (0.7)
Web page/Web site	–	–	–	–	74 (17.8)
Other	–	–	–	–	23 (5.5)

a 173 conference material, 12 meeting abstracts and 30 proceedings.

Searching citation databases is likely to result in the inclusion of non-relevant records in the intended output. For example, simply querying for “fibre” AND “forensic science” in the title, abstract and keywords with the aim to retrieve references related to garments and textiles crosses to other research topics and disciplines, such as physics with “fibre optics”, toxicology with “herb fibre” or medicine with “muscle fibres”. When unwanted references were excluded, the 1756 references obtained from the combined search on Scopus and Web of Science was reduced to 625 documents related to fibres associated with textiles and garments. The detailed results are included in Table 1. A total of 245 (39.2%) references were found to be exclusive to Scopus, 79 (12.6%) to Web of Science and 301 (48.2%) shared between databases.

**Fig. 1:**
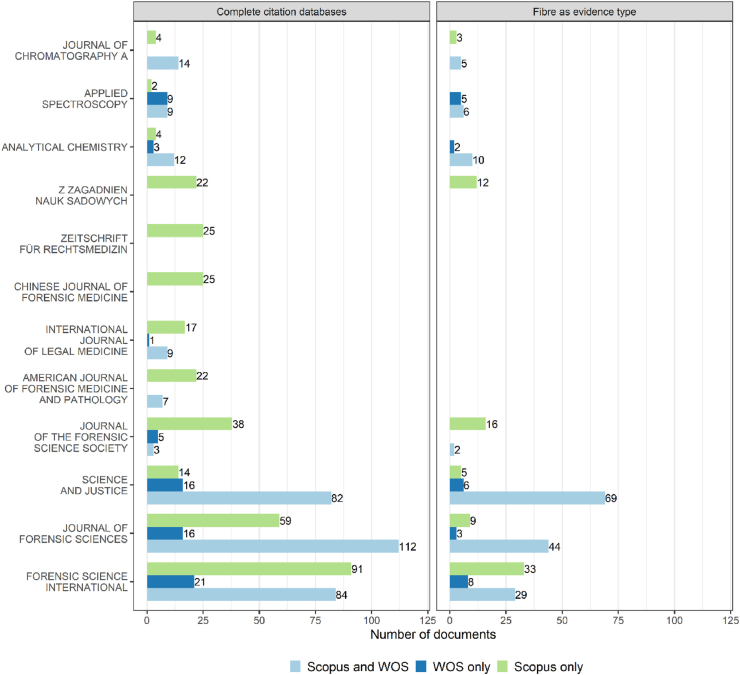
Number of outputs sorted by source title indexed only in Scopus, Web of Science and shared in both citation databases for the search criteria fibre and forensic science. Top 12 journal titles ordered by their overall total number. Left: All source titles are included prior to applying any exclusion criteria, and Right: Source titles for fibre as evidence type associated with textiles and garments. The original journal titles were retained, but when a journal changed its name the results could be combined with its previous title.

Documents published in “*Forensic Science International*” were the most indexed in Scopus with 175 entries out of 1569 (11.2%), while outputs published in “*Journal of Forensic Sciences*” were the most common for Web of Science (128 references out 867, 14.8%). Of the combined indexed materials in both Scopus and Web of Science, 112 (out of 674, 16.6%) were found to be published in the journal “*Journal of Forensic Sciences*”, 84 (12.5%) in “*Forensic Science International*” and 82 (12.1%) in “*Science and Justice*”. These observations confirm that there are certain forensic science journals which are well indexed in both citation databases but not necessarily equally represented, as illustrated in Fig. 1. As an example, for the 196 documents from “*Forensic Science International*” listed in the combined dataset generated from the Scopus and Web of Science outputs, 84 records are shared between the two citation databases while 21 were found to be exclusive to Web of Science and 91 only to Scopus. This also demonstrated that, while some journals are well represented in citation databases, literature searches did not systematically return all possible entries, showing the benefit of cross-referencing output data from multiple databases to generate a comprehensive dataset.

### 3.2 Keywords

**Fig. 2:**
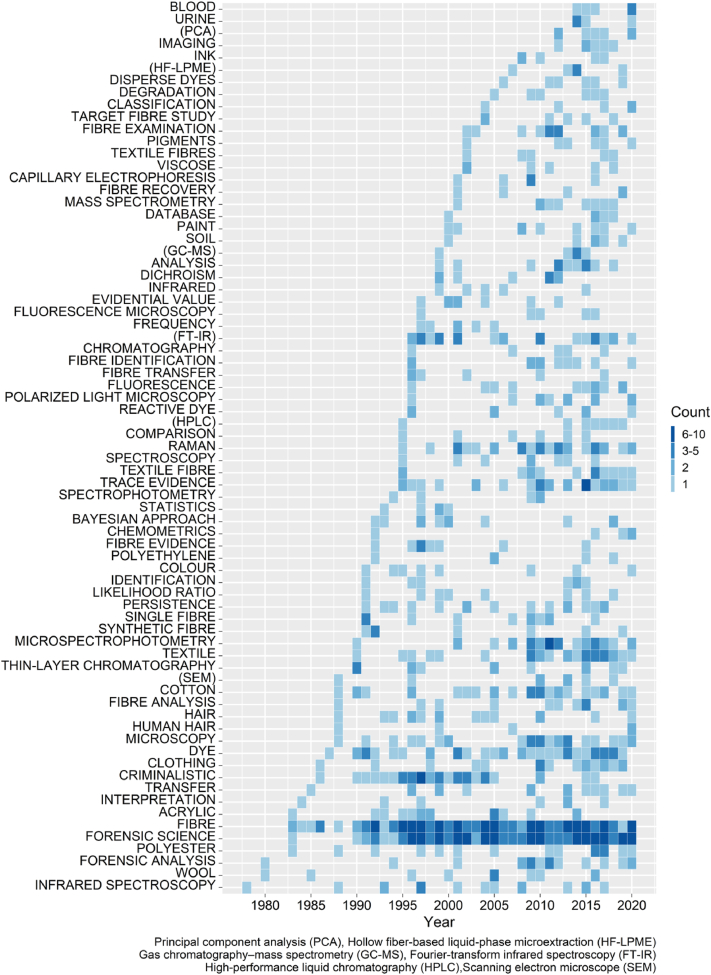
Most frequently used author's keywords in the dataset generated by Scopus and Web of Science on fibre related to textile and garments in forensic science. List of 73 keywords or acronyms ordered by their first year of appearance, minimum number of occurrences set to 5.

The 625 outputs from the combined dataset of Scopus and Web of Science contained 3099 author's keywords (AK), 1701 of which were distinct. 135 documents, mainly articles (66.6%), did not provide any author's keywords. The earliest records using author's keywords were from two articles published in 1978 by Hughes et al. and Yamamoto and Yamamoto [22,23]. A list of the most frequently occurring keywords is presented in Fig. 2. Keyword choice is seen to progress over time with the increase in the number of publications but also reflects changes in research interest.

The top 5 keywords with the highest frequency of appearance were “Fibre”, “Forensic Science”, “Criminalistic”, “Dye” and “(FT-IR)”. The keyword “Fibre” on its own was mentioned for the first time in 1983 in an article by Grieve entitled: “The use of melting point and refractive index determination to compare colourless polyester fibres” [24].

Textile fibres such as “Cotton”, “Polyester” and “Acrylic” are also listed in the most frequently occurring keywords presented in Fig. 2. This is in line with the textile industry where cotton is found to dominate the natural fibre market and polyester represents the majority of the chemical fibre production [25,26], hence reflecting the importance of these types of fibre in forensic science examination. The keywords listed in Fig. 2 also provided information on the most frequently employed techniques and type of analysis carried out, with the most used techniques being Raman Spectroscopy, Fourier-Transform Infrared Spectroscopy (FT-IR) and Microspectrophotometry (MSP), with 31 counts each. It is also noteworthy to see keywords such as “Blood”, “Paint” or “Ink” frequently being listed as keywords but they also reflect the scientific interest of the concerned literature since fibre evidence is often considered with other types of micro traces.

### 3.3 Authors

**Fig. 3:**
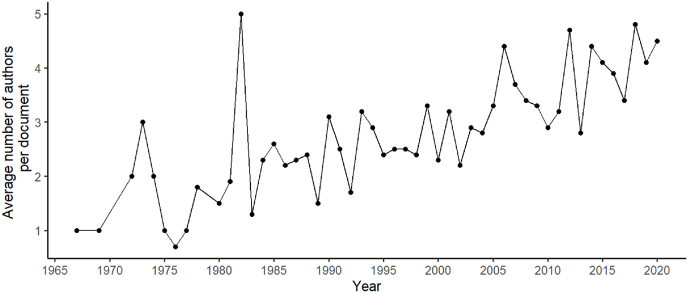
Representation of the average number of authors per document and year for the combined dataset of Scopus and Web of Science.

A total of 1417 distinct authors were retrieved from the 625 documents available from Scopus and Web of Science combined, with 1123 (79.3%) of authors having published only once in this area of research, and 294 (20.7%) authors having their names on multiple documents. The top publishing authors were found to be: Grieve, M.C., who was listed as an author in 29 publications (between 1983 and 2017), followed by Roux C.P. (22 publications, from 1996 to 2017) and Wiggins K.G. (19 publications, 1987 to 2017). The average number of authors per output has also been seen to increase over the years. Fig. 3 reveals this trend.

**Fig. 4:**
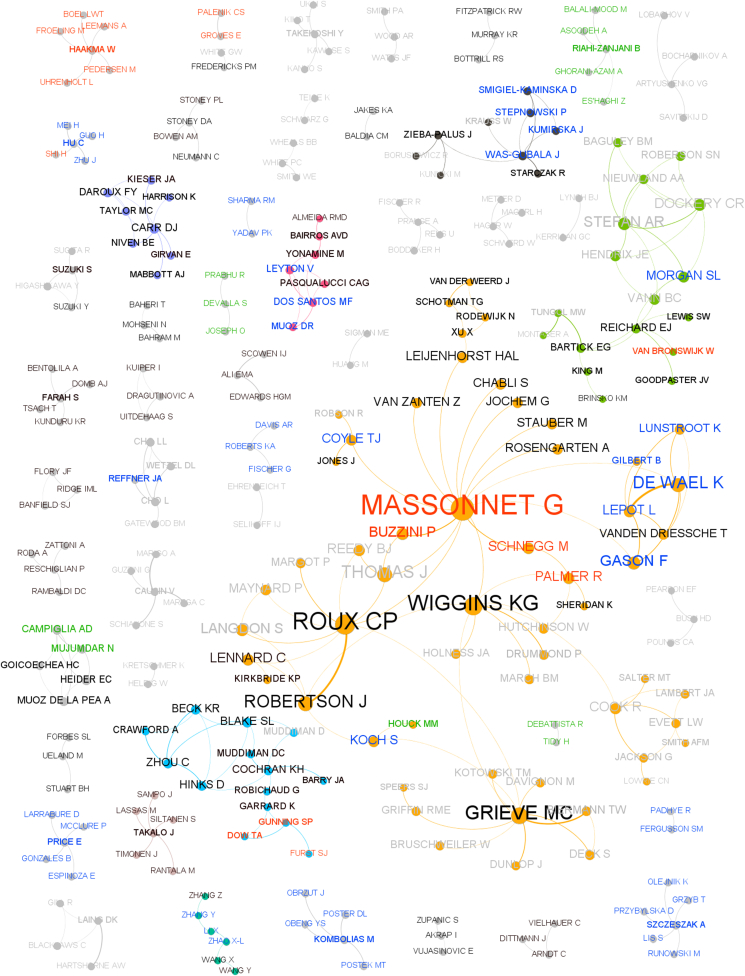
Co-authorship network between authors with multiple publications, generated by Gephi and ForceAtlas2 algorithm. Total of 269 authors. The nodes represent authors and the lines co-authorship. Eigenvector centrality was used to determine the size of the node. Each group of authors is colour-coded. The width of the line is proportional to the number of contributions between the two authors. The name of the authors who have their last publication in 2010 or later are colour-coded:2010–2017 in black, 2018 in red, 2019 in green, 2020 in blue. (For interpretation of the references to colour in this figure legend, the reader is referred to the Web version of this article.)

A co-authorship network out of the 294 authors whose names appear twice or more in the document list is shown in Fig. 4. Of the 294 authors, 25 authors published alone or with authors that only published once and therefore appeared not to be part of any collaborative network. As a result, 269 authors appear in Fig. 4.

The radius of the author's node and the font size of its label are proportional to their eigenvector centrality: a high eigenvector score (maximum value 1) signifies that a particular node is connected to many other nodes with high scores, a differentiation from in-degree centrality which refers instead to the number of receiving links by that node. The thickness of the line between two nodes is proportional to the number of interactions between the two nodes. The author's eigenvector centrality has a long tail distribution where only two authors had an eigenvector centrality greater than 0.7 and four authors greater than 0.5. Not all the nodes are connected to one another; the absence of bridging between authors means that authors predominantly published with members of their own research organisation or collaborative networks. There is one main cluster with 56 authors (orange), one medium cluster (green) with 17 authors and 45 smaller groups of authors. This demonstrates the number of authors on publications rather than the actual number of publications (Massonnet with 55 co-authors across 10 publications).

### 3.4 IFSMS reports

**Fig. 5:**
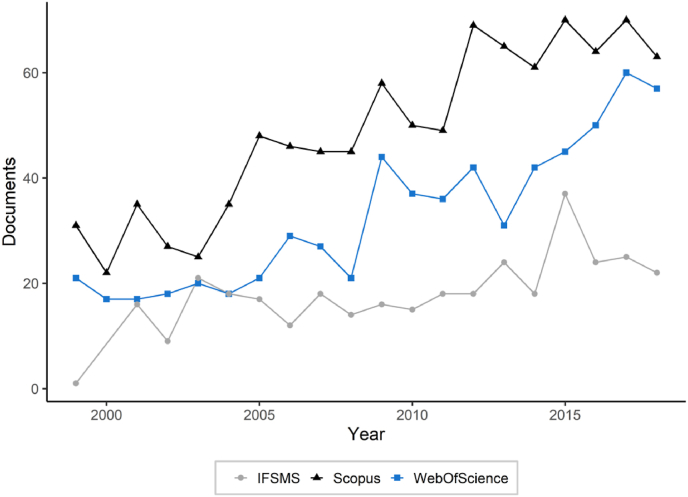
Publications in Fibre and Forensic Science from 1999 to 2018. The black line represents publications from a Scopus search, the blue line the publications from a Web of Science search and the grey line the publication from the IFSMS reports. (For interpretation of the references to colour in this figure legend, the reader is referred to the Web version of this article.)

A total of 417 distinct references were collated from the six latest IFSMS reports (2004–2019), Table 1. The majority of outputs were articles (n = 185; 44.4%), proceedings papers (n = 82; 19.7%), and websites (n = 74; 17.7%). Fig. 5 represents the yearly distribution of documents relating to fibres listed in the IFSMS reports, Scopus and Web of Science. 297 documents out of 417 (71.2%) appearing in the IFSMS were not listed in either Scopus or Web of science. The majority of these references were proceedings (n = 82) and webpages (n = 74), documents generally not indexed in the two citation databases.

**Fig. 6:**
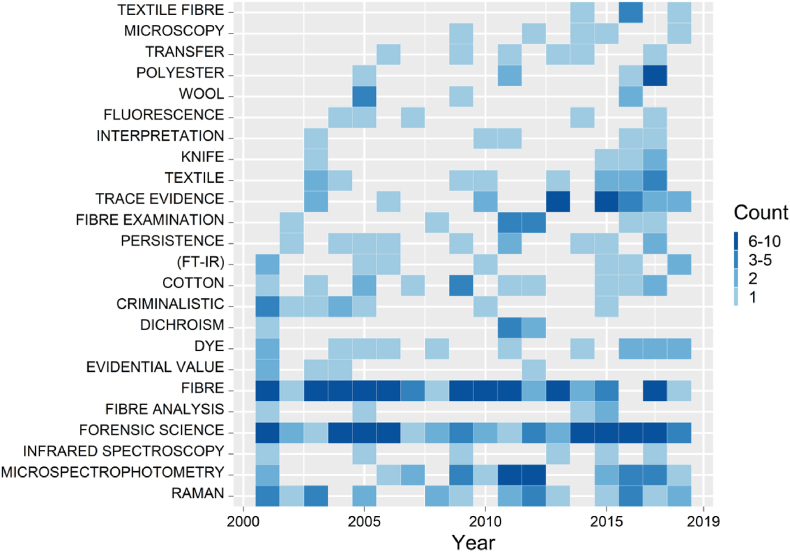
Most commonly found keywords in the IFSMS report, from 2004 to 2018. List ordered by first year of appearance. The minimum number of occurrences was set to five.

The 417 publications in the IFSMS report generated a total of 1029 author's keywords (AK) of which 618 were distinct. This is fewer than the number reported from the citation databases but expected given 227 outputs, for example websites, did not provide keywords. Keyword distribution for the IFSMS reports is presented in Fig. 6. “Fibre” was the most frequent keyword appearing in 71 references, followed by “Forensic Science” (n = 63), “Microspectrophotometry (MSP)” (n = 28), “Raman” (n = 25) and “Trace Evidence” (n = 21). As observed for the keywords from the citation databases, Fig. 2, cotton and polyester are the most common terms provided the authors, appearing 14 and 8 times, respectively, followed by wool (n = 6) and acrylic (n = 4).

Only three techniques are listed in Fig. 6. In addition to the previously mentioned “Microspectrophotometry (MSP)” and “Raman”, “Fourier-Transform Infrared Spectroscopy (FT-IR)” was also observed as a frequent keyword. Although this illustrated fewer techniques than the list provided by the citation databases, it is a simple reflection of the lower number of references and keywords from the references listed in the IFSMS reports.

A total of 940 authors (592 distinct) were retrieved from the six IFSMS reports and their 417 references, with 473 (79.9%) authors having only one publication and 119 (20.1%) appearing on multiple documents. Roux C.P. had the highest number of publications (n = 28) followed by De Wael K. (n = 19) and Robertson J. (n = 19). These observations were complementary to those of the citation databases, in which Roux C.P. was also found to be listed as one of the authors contributing the most to the field. The difference between the IFSMS and the citation databases author lists can be explained with a better match between the year range of the IFSMS report (i.e. 1999 to 2018) and publication activity for Roux C.P.

## 4 Discussion

The inclusion of information from more than one database, in this case the aggregation of outputs indexed on Scopus and Web of Science, as well as their filtering to exclude records thought to be non-relevant, all combined with reference lists provided by the INTERPOL review documents, allows the easy generation of a specific list of references connected to the sought topic of research. The comparison between Scopus and Web of Science, focussed on forensic science and fibres as in clothing and garments.

The analysis showed that there is an overlap of 48.2% (n = 301 out of 625) within the indexed records listed in the two citation databases, this observation is similar to findings in studies covering other topics [7,9], emphasising the benefit of combining information from various sources to get the most comprehensive list of outputs. On the other hand, with such an overlap between the list of references given by Scopus and Web of Science, combined with the financial saving of accessing and the simplicity of dealing with outputs of only one database, it would be reasonable to expect forensic scientists and researchers can collect significant literature information using just one citation database.

Familiarity with a given platform could be a reason for such a decision, but also possibly as a consequence of financial constraints in using subscription-based information. In the absence of access to Scopus or Web of Science, it was interesting to observe that a significant proportion (35.8%, n = 224 out of 625) of forensic research publications on fibres from textile and garments were available in four journals: Science and Justice (n = 80 records), Forensic Science International (n = 70 records), Journal of Forensic Sciences (n = 56 records) and Journal of the Forensic Science Society (prior the journal being renamed Science and Justice, n = 18 records). These journals are multidisciplinary covering a broad range of topics, the browsing of which would lead to significant information capture. Extensive literature coverage may still be achieved by cross-referencing citations, a practice generally followed by the community regardless of the approach and initial choice. Another approach is the use of information disseminated by social networks. While social networks may not provide access to documents unless they were published Open Access, social networks can rapidly report on the latest development. They are also a great source of information for research and other materials that could neither be published in forensic science journals nor found indexed in citation databases.

In the IFSMS reports, 32.4% (n = 135 out of 417) of the listed references were indexed in Scopus and/or Web of Science, and only 28.8% (120 out of 417) of those matched outputs by the citation database following a specific search (reduced dataset based of fibres associated with textiles and garments). These results were found similar to observations made for other evidence types listed in the IFSMS reports [12] for which 3216 out of 14,718 (21.9%) references listed across 13 evidence types and indexed on Scopus were found also present in targeted searches relevant to the individual evidence types, for example toxicology, explosives, glass or paint. This disparity may seem surprising since the IFSMS reports have the purpose of communicating the latest scientific discoveries from the previous three years, with one reasonable source being from the recorded information contained in citation databases. The IFSMS reports did not claim to be comprehensive on the topic, and as they were written by forensic practitioners, they could be interpreted as a means whereby experts of the subject matter could identify gaps within specific areas.

The authors of the IFSMS reports decided on the content of the reviews with a requirement to focus on the outputs that have appeared in the previous three years. The reports aim to discuss the current problems, identify trends and their potential impact on forensic science, and offer possible solutions. With these objectives in mind, the authors may preferably include specific types of publications, for example, a significant proportion of outputs referred to within the INTERPOL review arose from conference meetings and abstracts. This decision may be encouraged by the typical audience reading the IFSMS reports, as to summarise the discussion between practitioners attending various working groups and conferences, but also to provide an account to anyone unable to attend such meetings. Equally, presenting the latest scientific activity and interest within the community could be seen as a platform to encourage further discussion, future collaboration, and growth in scientific discoveries via forthcoming publication.

Some proceedings reported in the IFSMS reports were later developed as peer-reviewed articles. For example, Massonnet and Buzzini presented their research in 2008 at the 16th European Fibres Group Meeting in Budapest (Hungary) on the discrimination of coloured acrylic, cotton, and wool fibres using Raman spectroscopy, (proceedings described in the 16th (2010) IFSMS report) before their work was published in 2013 [27] and 2015 [28]. This highlights the importance of including early work discussed in conference proceedings, as well as case study discussions, in the IFSMS reports. As well as providing solutions, case studies may also highlight gaps in scientific knowledge and encourage further research in specific areas.

The authors of fibres and textiles sections in the INTERPOL reports regularly highlighted the need for the development of a database of fibres, with searchable properties and data. Access to such information would assist casework in terms of intelligence gathering as well as potentially allowing the delivery of more robust interpretations and conclusions. This vision of the IFSMS authors to report on the need to develop a database for fibres and other evidence types is interesting as it differs from the trends observed in the records indexed in Scopus and Web of Science. For example, author's keywords, Fig. 2, gives an indication of the past research interest (recent or more distant).

However, even though “Database” is a frequently used author's keyword, particularly since 2016, very few documents have a dataset attached to their records. In this work, the survey of over 160 records generated by Web of Science found just one document [29] with its dataset placed in a repository [30] and accessible via a digital object identifier (DOI). Interestingly, even research articles which focused on the creation of databases failed to provide information where the discussed data could be found [31,32]. Publishing data is the public disclosure of the collected research data and makes the research data retraceable for verification purposes and reusable for future research. Furthermore, data sharing allows the information to be used as a reference in other studies, to cross-validate methods and work. In so doing, sharing data can lead to the development of new collaborative networks, further expanding the reach and impact of the research. Such collaboration could come from other scientists with similar research interests but also from other disciplines. For example, in the case of fibre as an evidence type, interests may come from fashion, clothing and textile research in the discipline of Arts, Humanities and Social Sciences. Finally, a point that is generally omitted as a positive argument, data sharing may lead to the creation of collaborative ground-truth data sets incorporating information from one or more sources which has a certain significance and importance in forensic science. However, for this to be successful, the data must be provided in an agreed format, or a format which can be converted to a generic format and made openly accessible.

Data is not simply the results presented in the publication or the extra material included in the supplementary information (often given as Excel files with embedded figures, Word documents, PDF, etc), but all the files collected during data acquisition and converted in a machine-readable format. This could include, for example, images saved as a tagged image file format (TIFF), FT-IR spectra exported from the instrument as comma-separated values (csv), etc., which combined will form the dataset: a structured collection of files and metadata detailing its content. With research data policies now changing so that all research data underlying scientific publication must be made available for reuse, as well as many funders and publishers now encouraging such practice, the forensic science community should seize this opportunity to lead the way in making its data available.

As observed in this work, datasets generated following the search of the citation databases (i.e. Scopus and Web of Science) contain a large variety of fields such as title, authors, source title, year, affiliations, keywords etc., but there are currently very few, if any, entries related to data and its location in a repository. Citation databases have implemented various impactful changes over the years but the focus primarily remains on research metrics and citations. Without the means to automatically record data information as part of the extended reference details, it is left to the authors to specify its location in the manuscript. This information can be provided by making a simple and clear statement in the data availability section or the methodology part of the manuscript, similar to the one made in this article. This situation is however far from ideal since it does not automatically make the data publicly available, especially if the manuscript is not published Open Access. It is therefore equally important that institutions hosting records on their research portal promote information regarding the associated datasets in conjunction with the record of publications to further assist data sharing.

## 5 Conclusion

The present study further revealed that the scientometric approach is a useful tool for identifying trends in fibres in forensic science but also identifying gaps in knowledge to help scientists create new research projects. The combination of references from two databases (i.e., Scopus and Web of Science) demonstrated an important overlap in the particular topic but also revealed differences in indexing, as for example some forensic science journals were indexed in both citation databases but not necessarily equally represented. The results showed that cross-referencing output data from multiple databases is of benefit in generating a comprehensive dataset.

A comparison between the IFSMS and the combined lists from Scopus and Web of Science revealed that only a limited number of documents were shared between the two generated datasets. Where the IFSMS reports have the purpose of communicating the latest scientific trends across forensic science evidence types from the previous three years, the data outputs were found to be mostly absent in both citation databases. The content of the IFSMS reports can be seen as complementary to the information available in other citation databases.

With an ever-increasing level of information becoming available, there is also a need to better promote data sharing. The forensic science community has the opportunity to lead the way in making their data available to their practitioner communities generating new collaborative networks and further expanding the reach and impact of their research.

## CRediT authorship contribution statement

**Virginie Galais:** Methodology, Software, Writing – original draft, preparation. **Holly Fleming:** Writing – original draft, preparation. **Niamh Nic Daéid:** Conceptualization, Funding acquisition, Writing – original draft, preparation. **Hervé Ménard:** Conceptualization, Software, Methodology, Writing – original draft, preparation.

## Declaration of competing interest

The authors declare that they have no known competing financial interests or personal relationships that could have appeared to influence the work reported in this paper.
